# Noninvasive Low-Intensity Focused Ultrasound Mediates Tissue Protection following Ischemic Stroke

**DOI:** 10.34133/2022/9864910

**Published:** 2022-07-04

**Authors:** Alexandra M. Kaloss, Lauren N. Arnold, Eman Soliman, Maya Langman, Nathalie Groot, Eli Vlaisavljevich, Michelle H. Theus

**Affiliations:** ^1^Department of Biomedical Sciences and Pathobiology, Virginia Tech, Blacksburg, VA 24061, USA; ^2^Department of Biomedical Engineering and Mechanics, Virginia Tech, Blacksburg VA 24061, USA; ^3^Center for Engineered Health, Virginia Tech, Blacksburg Virginia 24061, USA

## Abstract

*Objective and Impact Statement*. This study examined the efficacy and safety of pulsed, low-intensity focused ultrasound (LIFU) and determined its ability to provide neuroprotection in a murine permanent middle cerebral artery occlusion (pMCAO) model. *Introduction*. Focused ultrasound (FUS) has emerged as a new therapeutic strategy for the treatment of ischemic stroke; however, its nonthrombolytic properties remain ill-defined. Therefore, we examined how LIFU influenced neuroprotection and vascular changes following stroke. Due to the critical role of leptomeningeal anastomoses or pial collateral vessels, in cerebral blood flow restoration and tissue protection following ischemic stroke, we also investigated their growth and remodeling. *Methods*. Mice were exposed to transcranial LIFU (fundamental frequency: 1.1 MHz, sonication duration: 300 ms, interstimulus interval: 3 s, pulse repetition frequency: 1 kHz, duty cycle per pulse: 50%, and peak negative pressure: -2.0 MPa) for 30 minutes following induction of pMCAO and then evaluated for infarct volume, blood-brain barrier (BBB) disruption, and pial collateral remodeling at 24 hrs post-pMCAO. *Results*. We found significant neuroprotection in mice exposed to LIFU compared to mock treatment. These findings correlated with a reduced area of IgG deposition in the cerebral cortex, suggesting attenuation of BBB breakdown under LIFU conditions. We also observed increased diameter of CD31-postive microvessels in the ischemic cortex. We observed no significant difference in pial collateral vessel size between FUS and mock treatment at 24 hrs post-pMCAO. *Conclusion*. Our data suggests that therapeutic use of LIFU may induce protection through microvascular remodeling that is not related to its thrombolytic activity.

## 1. Introduction

Ischemic stroke is a leading cause of death and disability that is often associated with a minimal degree of functional restoration. In the hyperacute phase of ischemic stroke, surgical thrombectomy following large vessel occlusion (LVO) is the optimal intervention to remove the primary obstruction and restore cerebral blood flow (CBF) to the penumbra [1, 2]. Because systemic treatment with IV rt-PA has shown little effect in the treatment of LVO, additional means of therapeutic intervention are needed to compliment current treatment options either prior to and/or following thrombectomy. Focused ultrasound (FUS) has been shown to provide good skull penetration, enhance thrombolytic effects, and reduce cerebral infarction in rodent studies when applied under a range of parameters directly or in combination with t-PA or other therapeutics [3–5]. However, unexpected hemorrhagic complications have been observed in clinical trials testing transcranial low-frequency ultrasound-mediated thrombolysis in brain ischemia (TRUMBI) trial, in which unfocused 300 kHz ultrasound pulses were applied using long pulses [6, 7]. This trial was ultimately stopped before completion due to high rates of hemorrhage in patients, likely due to the low ultrasound frequency and long pulses used in these treatments. These findings suggest that additional testing for optimal energy levels under cerebral ischemic conditions is needed.

Ultrasound (US) is known to have several biological effects depending on its emission characteristics. Recently, there is growing evidence that US at lower intensities (<2 W/cm^2^) facilitates enzymatic mediated thrombolysis by breaking fibrin polymers which can increase the effectiveness of thrombolytic drugs. However, in addition to its perceived thrombolytic effects, more information is needed to improve our understanding of the mechanistic underpinnings that may drive acute neuroprotection. Notably, there are no reports in the preclinical literature about intracerebral bleeding or relevant cerebral cellular damage at energy levels up to 1 W/cm^2^. Additionally, the emitted US beam widens with decreasing frequency, insonating increased volumes of intracerebral vasculature with the low-frequency US therapy and potentially damaging off-target tissues. Therefore, additional studies are needed to confirm the safety and efficacy of US therapy for stroke. Among several ultrasound technologies, low-intensity focused ultrasound (LIFU) has emerged as a noninvasive therapy for several diseases. Previous studies have shown this therapy upregulates neurotrophins, including VEGF and endothelial nitric oxide synthase (eNOS), in animal models of heart disease, vascular dementia, and Alzheimer’s disease ([8, 9]; S. H. [10]). Moreover, additional long-term functional benefits have been shown in models of stroke, with changes suggested to be mediated through angiogenesis and neurogenesis [11, 12].

In the present study, we examined the use of LIFU for the treatment of ischemic stroke using a 1.1 MHz single element FUS transducer designed to deliver transcranial LIFU in a murine model. Specifically, we evaluated whether directed LIFU could promote acute neuroprotection in a nonthrombotic murine model of permanent middle cerebral artery occlusion (pMCAO). Another important objective of this study was to verify the absence of adverse effects of LIFU (e.g., hemorrhage) on the brain under ischemic conditions. First, a pilot experiment was conducted to assess the safety of the LIFU therapy applied under two peak negative pressures conditions: ~2.0 MPa and 3.5 MPa. Then, additional experiments were completed at a peak negative pressure of 2.0 MPa to determine a mechanism of action unrelated to thrombolytic effects. The efficacy and safety of LIFU under the experimental ultrasonic conditions for ischemic stroke therapy were evaluated using a series of postmortem analyses, while closely monitoring for adverse effects of FUS treatment (e.g., hemorrhage) on the brain under ischemic conditions.

## 2. Results

### 2.1. Transcranial LIFU Safety and Efficacy

**Figure 1:**
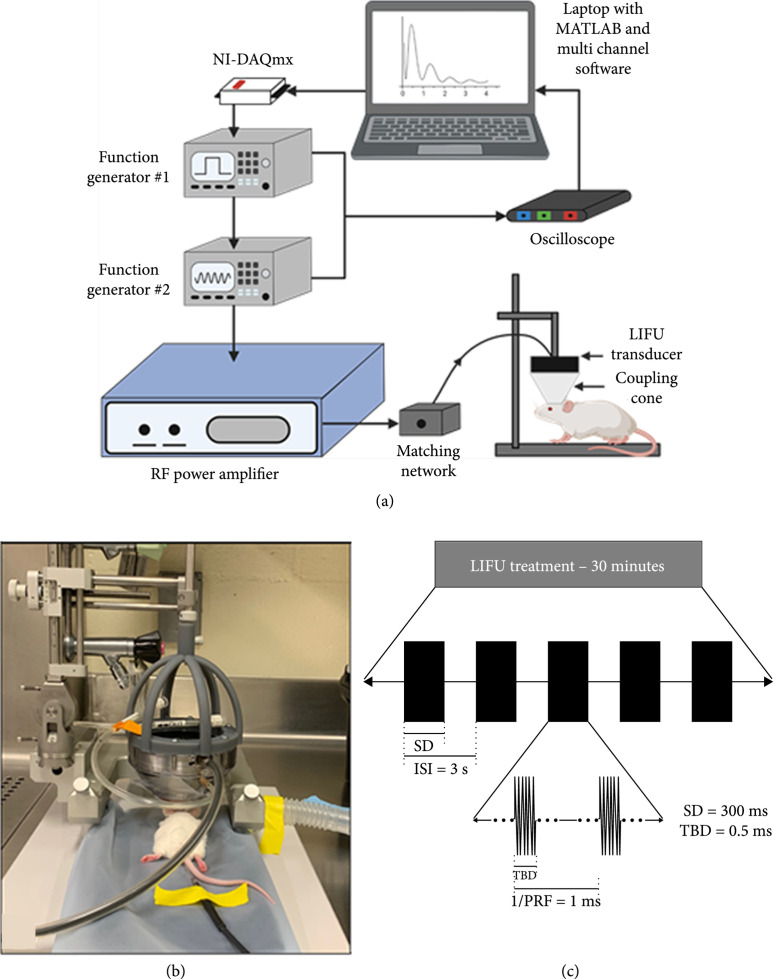
Experimental setup for focused ultrasound treatment following ischemic stroke. (a) Two connected function generators were driven by a custom MATLAB computer program to generate a pulsing signal amplified by a linear power amplifier and transmitted to the FUS transducer. (b) Transcranial focused ultrasound was applied to the cerebral vasculature of an adult, male CD1 mouse using a 1.1 MHz single element transducer coupled to the skull with a coupling cone filled with degassed water. (c) Mice were treated with LIFU at a peak negative pressure of either -1.8 MPa, -2.0 MPa, or -3.5 MPa and PRF=1 kHz, SD=300 ms, ISI=3 s, and TBD=0.5 ms for 30 minutes. (a) was created in part with http://Biorender.com.

**Figure 2:**
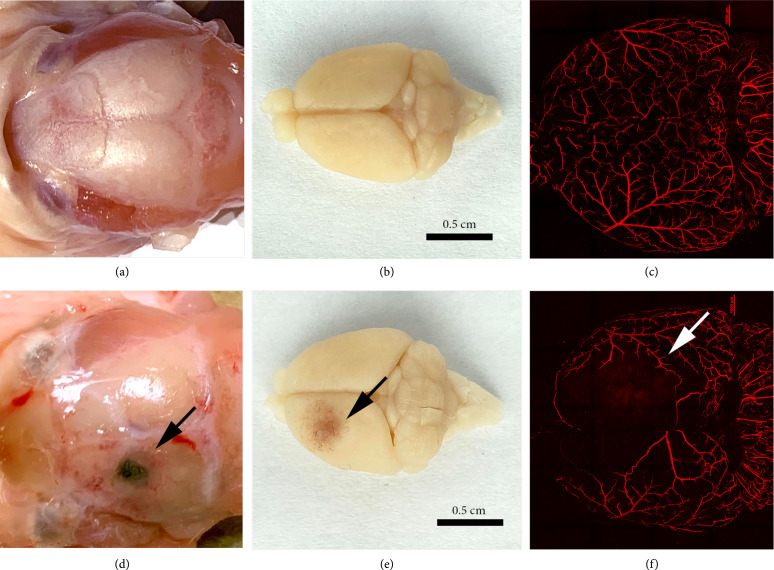
Atypical tissue damage and mortality observed after LIFU treatment at *p*− 3.5 MPa. (a, b) Representative images of nonhemorrhagic sham brain after LIFU treatment at *p*− 1.8 MPa. (c) Example of a vessel-painted confocal image after sham surgery and LIFU treatment without ultrasound-induced damage at *p*− 1.8 MPa. (d, e) Representative images of hemorrhage observed on the brain surface of sham animal after LIFU treatment at *p*− 3.5 MPa (arrow). (f) Example of a vessel-painted confocal image of tissue damage after sham surgery and LIFU treatment at *p*− 3.5 MPa (white arrow). Scale=1000 *μ*m.

LIFU was applied transcranially for 30 minutes using a 1.1 MHz single element FUS transducer following pMCAO for all groups (Figure 1). To determine the safety of transcranial LIFU under the experimental ultrasonic parameters, a pilot study was first conducted comparing two peak negative pressures (*p*−) (1.8 MPa and 3.5 MPa). Sham or pMCAO (i.e., stroke) adult, male CD1 mice in the pilot group were exposed to 30 minutes of LIFU at a peak negative pressure of either 1.8 MPa or 3.5 MPa, with an ultrasonic pulse applied every three seconds ($\text{interstimulus}\;\text{interval}\;\left(\text{ISI}\right)=3$ s) (n=3‐4 mice per group). A pulse repetition frequency (PRF) of 1 kHz, duty cycle (DC) per pulse of 50%, sonication duration (SD) of 300 ms, and tone-burst duration (TBD) of 0.5 ms were used in all experiments. Throughout the surgery and LIFU treatment, the animal was anesthetized using isoflurane and monitored for signs of distress or changes in temperature and respiration rate. After LIFU treatment, the animal’s behavior was noted during recovery, and the treated region was monitored for hemorrhage. Mortality occurred in one of three sham mice and one of four pMCAO mice in the 3.5 MPa treated group. Additionally, hemorrhaging occurred in one of two remaining sham mice and one of three remaining pMCAO mice treated with 3.5 MPa LIFU (Figures 2(a) and 2(b) and 2(d) and 2(e)). The destruction of cerebral arteries and connecting pial arteries was appreciable in the vessel-painted images in the area of 3.5 MPa LIFU treatment where hemorrhaging occurred (Figures 2(c) and 2(f)). No mortality or hemorrhage was noted in the sham or pMCAO groups treated with 1.8 MPa LIFU. Based on the results from the pilot study, a peak negative pressure of 2.0 MPa was chosen for neuroprotection experiments to maximize the potential effects of the LIFU treatment while minimizing any risk to the subjects.

### 2.2. LIFU Prevents Cortical Tissue Damage and BBB Permeability following pMCAO

**Figure 3:**
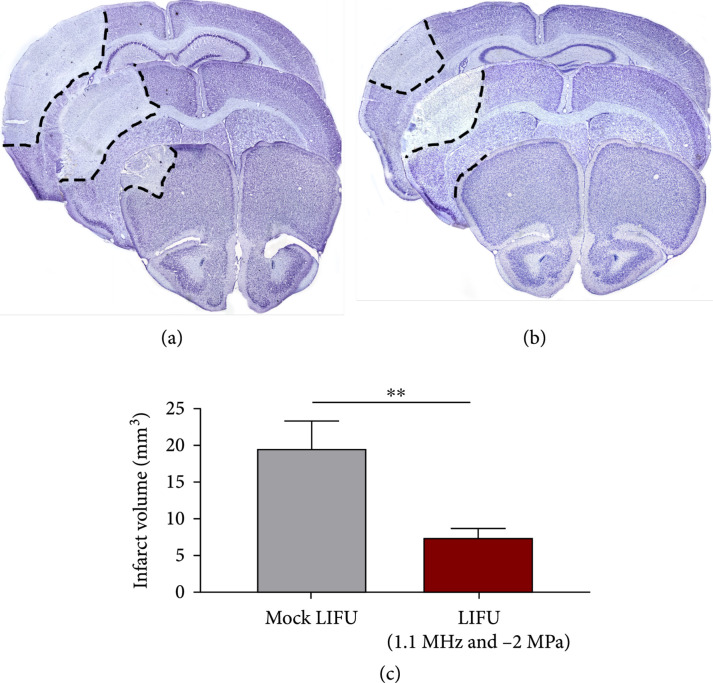
Infarct volume reduced in mice treated with LIFU following ischemic stroke. (a) Representative images of serial Nissl-stained sections at 3 bregma levels in mock-treated and (b) LIFU-treated mice, 1-day post-pMCAO. (c) Quantified data shows a significant reduction in infarct volume in FUS-treated mice compared to mock-treated controls; n=8‐9. ${}^{**}P<0.005$.

**Figure 4:**
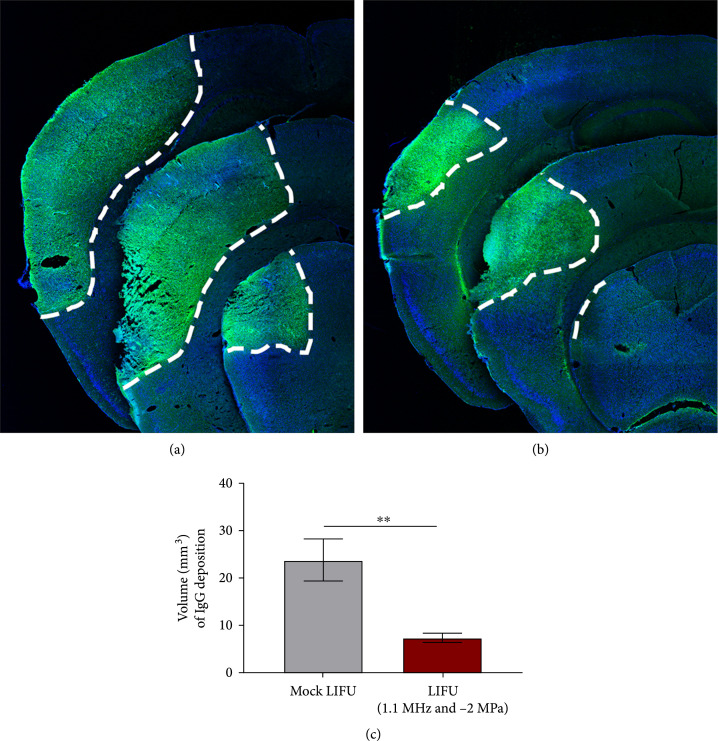
IgG deposition reduced in LIFU-treated mice after pMCAO. (a) Representative confocal images of sections immunolabeled with anti-IgG (green) and counterstained with DAPI (blue) from mock-treated and (b) LIFU-treated mice 1-day after pMCAO. (c) Quantified analysis showing reduced deposition of IgG in LIFU-treated animals compared to mock-treated controls. n=5‐7. ${}^{**}P<0.005$.

Utilizing the pMCAO model of ischemic stroke [13], adult male CD1 mice were first subjected to stroke surgery followed by 30-minute exposure to 1.1 MHz, 2.0 MPa peak negative pressure LIFU, or mock LIFU. In the mock LIFU group, the mice were still placed under the transducer and exposed to isoflurane for 30 minutes but did not receive any ultrasound treatment. As seen in the pilot experiment, mice were maintained at 37±0.5°C and were monitored for changes in respiratory rate or signs of distress. No distress, changes in respiration rate, or hemorrhage were noted in any of the mice. Mice were recovered for 24 hours (hrs) then were euthanized and evaluated for infarct volume. Using 6 Nissl-stained coronal serial sections, we quantified the infarct volume using the Cavalieri probe in MBF StereoInvestigator [14] and observed that mice receiving the LIFU therapy showed a significant reduction in infarct volume compared to mock LIFU (7.51±1.44 mm^3^ vs. 19.62±3.68 mm^3^, respectively, n=8‐9 per group ${}^{**}P=0.005$) (Figures 3(a)–3(c)). Next, we stained serial coronal sections with anti-mouse-488 antibodies to identify and quantify the area of IgG deposition in the brain at 24 hrs post-pMCAO. Compared to mock LIFU (23.77±4.43 mm^3^, n=5), we observed a significant reduction in LIFU-treated mice (7.29±2.56 mm^3^, n=7, ${}^{**}P=0.0016$) (Figures 4(a)–4(c)). IgG deposition was seen in anterior to posterior ipsilateral cortical hemisphere of mock-treated pMCAO mice (Figure 4(a)) which was substantially attenuated in LIFU-treated mice (Figure 4(b)).

### 2.3. LIFU Increased Microvessel Size in the Ischemic Cortex following pMCAO

**Figure 5:**
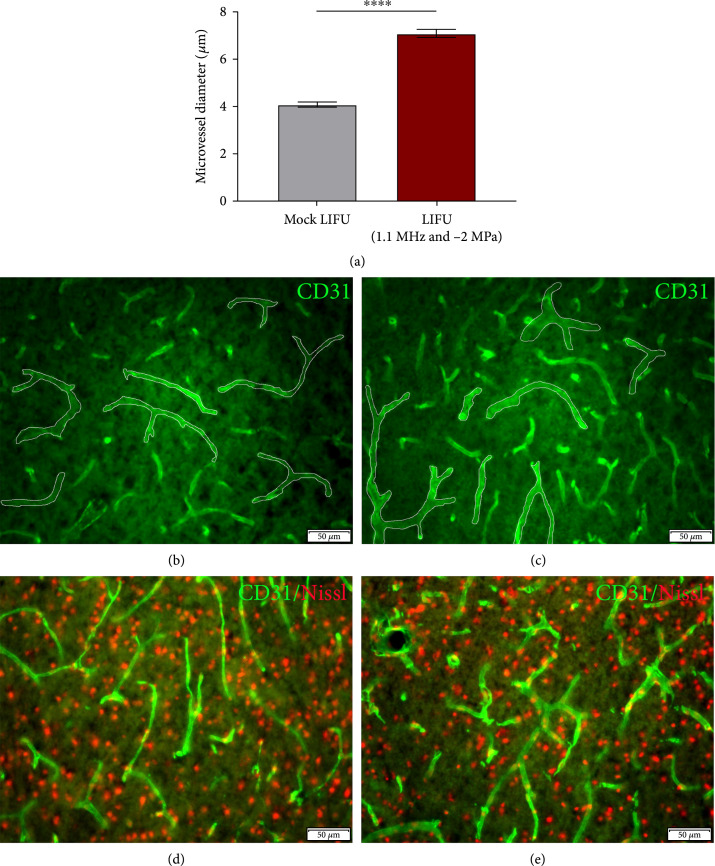
Microvessel size increased in mice treated with LIFU following ischemic stroke. (a) Quantified analysis showing increased microvessel diameter in LIFU-treated animals compared to mock-treated controls. n=4‐5. ${}^{****}P<0.0001$. (b–e) Representative images for IHC staining of CD31 (microvessels, green) and Nissl (neurons, red) in the ipsilateral cortex of (b, d) mock-treated and (c, e) LIFU-treated mice 1-day after pMCAO.

To determine if LIFU-mediated neuroprotection is correlated with the microvascular remodeling, we stained coronal sections with a vascular marker, CD31, and measured the microvessel diameter in the ipsilateral cortex. We found that LIFU treatment significantly increased the vessel diameter (7.07±0.17 *μ*m, n=5) when compared to mock control (4.06±0.11 *μ*m, n=4) (Figures 5(a)–5(e)). This observation suggests a vasodilatory effect of ultrasound which may contribute to the protection against pMCAO-induced ischemic stroke.

### 2.4. No Change in Leptomeningeal Anastomoses in Response to LIFU Treatment following pMCAO

**Figure 6:**
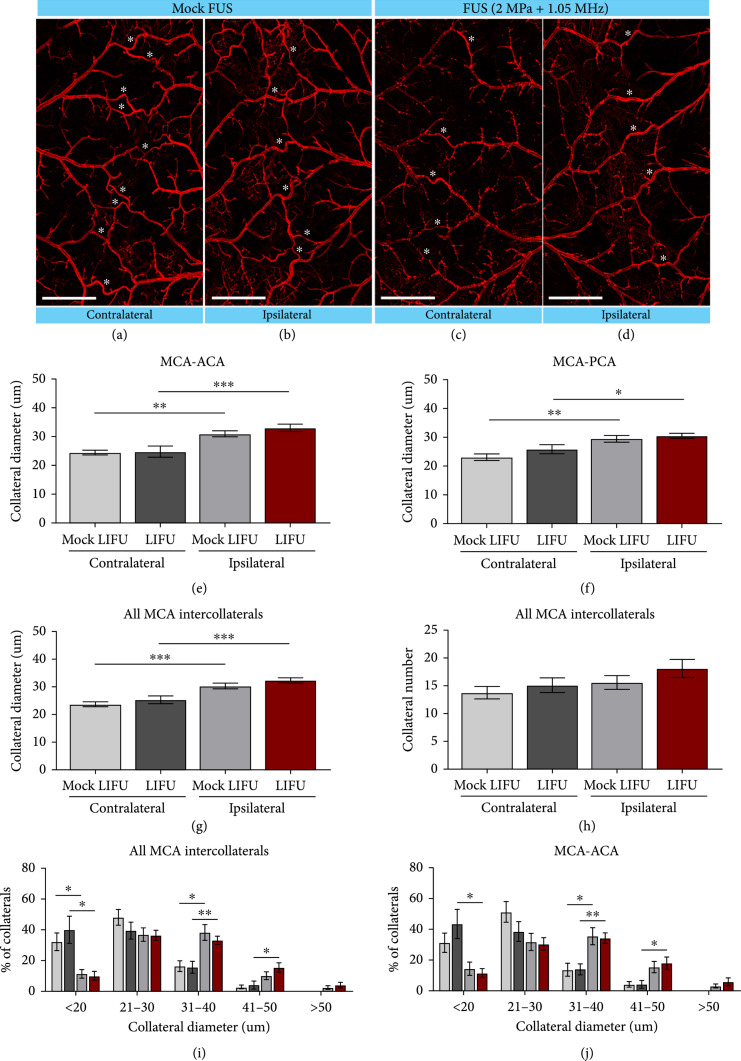
LIFU treatment does not significantly alter pial collateral size. (a, b) Representative vessel-painted confocal images of pial collaterals (stars) in both mock LIFU and (c, d) LIFU-treated brains 1-day post-pMCAO. (e) MCA-ACA, (f) MCA-PCA, and (g) average MCA intercollateral diameters 1-day post-pMCAO. Ipsilateral collaterals were significantly larger than the contralateral collaterals in both groups. No significant difference was seen in collateral diameter size between mock- and LIFU-treated groups. (h) Intercollateral counts show no significant difference in the number of pial collaterals in the control and treated groups. (i) Breakdown of MCA intercollateral and of (j) MCA-ACA collateral diameters after pMCAO. n=11‐12. ${}^{*}P<0.05$; ${}^{**}P<0.005$; ${}^{***}P<0.001$.

Leptomeningeal anastomoses or pial collateral vessels are *anastomotic* vessels that provide alternative routes for retrograde cerebral blood flow into the occluded territory following stroke and have a role in preserving the penumbral tissue. Due to the proximity of these vessels to the cranial LIFU treatment, we evaluated whether tissue protection may be due, in part, to their functional remodeling. Using our established vessel painting technique [13, 15, 16], we sought to quantify the diameter and number of pial collaterals at 24 hrs post-pMCAO in the ipsilateral and contralateral hemispheres of mock LIFU (n=12) and LIFU-treated mice (n=11). As we previously demonstrated [13, 16], we observed a significant increase in the diameter (*μ*m) of ipsilateral MCA-ACA intracollaterals (30.98±3.75 *μ*m) compared to contralateral (24.44±2.80 *μ*m; P=0.0039) in the control mock FUS mice (Figures 6(a), 6(b), and 6(e)). Similarly, we found LIFU-treated (-2 MPa, 1.1 MHz) mice also showed a significant increase in collateral size in the ipsilateral (33.04±4.26 *μ*m) vs. contralateral (24.75±6.24 *μ*m, P=0.004) hemispheres (Figures 6(c)–6(e)); however, no difference was observed between mock- and LIFU-treated mice. These changes were also observed in the MCA-PCA collaterals (Figure 6(f)) and in all MCA intercollaterals (Figure 6(g)). No difference in collateral number was seen across groups (Figure 6(h)). Lastly, the size distribution for pial collaterals was assessed. For all MCA intercollaterals (Figure 6(i)), we observed an increase in collateral size in the ipsilateral hemisphere of LIFU-treated mice, with 33.2% of collaterals ranging from 31 to 40 microns and 15.7% from 41 to 50 microns compared to 15.9% and 4.25% in the contralateral hemisphere, respectively. Conversely in mock LIFU mice, a significant increase was seen only in the 31-40-micron range, which contained 38.4% of ipsilateral collaterals but only 16.5% of collaterals in the contralateral hemisphere. Both mock- and LIFU-treated mice had significantly fewer collateral vessels ranging less than 20-micron range on the ipsilateral hemisphere (mock: 11.6% and LIFU: 10.2%), compared to the contralateral (mock: 32.4% and LIFU: 40.2%). For pial collaterals connecting the MCA-ACA (Figure 6(j)), the previous trends for LIFU-treated mice remain unchanged with significantly more ipsilateral pial collaterals in the 31-40 and 41-50 range (34.2% and 17.9%) compared to contralateral collaterals (14.9% and 4.2%). Significantly more ipsilateral collaterals were only observed in 31-40-micron range for the mock LIFU group (Ipsi: 35.4% and Contra: 13.6%). Lastly, the LIFU-treated mice also had significantly fewer ipsilateral collaterals in the <20-micron range, with 14.3% of ipsilateral and 31.2% of contralateral collaterals in this range. Although no significant changes were seen in pial collateral size in the LIFU-treated group at 24 hrs, it does not rule out the ability for LIFU treatment to improve or alter vasodilation within the pial collateral niche, which could influence neuroprotection. Overall, these findings suggest LIFU may offer significant neuroprotection through alternative mechanisms to arteriogenesis.

## 3. Discussion

The current study provides further evidence of the safety and neuroprotective properties of LIFU in a murine model of pMCAO. We observed a ~2.5-fold decrease in the infarct volume induced by pMCAO at 24 hrs post-pMCAO, and this result correlated with reduced area of IgG deposition in the ipsilateral cortex. Vasogenic edema is a major concern following ischemic stroke and is due to the disruption of the blood-brain barrier (BBB). The decrease in IgG deposition observed indirectly measures BBB function and suggests LIFU may function to prevent the accumulation of extracellular fluid. A decrease in edema following surgically induced ischemic stroke with LIFU treatment has been shown by multiple groups, further supporting this hypothesis [17–19].

We further demonstrate that microvascular remodeling may influence LIFU-mediated protection. LIFU-treated mice showed a significant increase in CD31-positive vessel diameter, which may indicate acute vasodilatory effects of ultrasound. Indeed, previous findings in humans show noninvasive transcutaneous low-frequency ultrasound causes vasodilation in brachial arteries [20]. Focused ultrasound has been shown to trigger the endothelium to release nitric oxide resulting in dilation of blood vessels [21]. Activation of the endothelial cells by focused ultrasound treatment supports VEGF signaling, angiogenesis, and restoration of the BBB, most notable under the anesthetic isoflurane, which was used in the present study [22]. These changes may play a central mechanistic role in the neuroprotective properties of LIFU under ischemic stroke conditions. The sustained vasodilation in the microvessels may also explain, at least in part, the increase in cerebral blood flow by roughly 20% seen in other studies at least 30 minutes posttranscranial focused ultrasound treatment [19]. Transcranial LIFU was applied to the cerebral vasculature, including the MCA-ACA pial collateral niche, after ischemic stroke using a 1.1 MHz single element transducer. Pulsed LIFU was delivered to the affected cerebral tissue at a PRF of 1 kHz, a duty cycle per pulse of 50%, a sonication duration of 300 ms, and an interstimulus interval of 3 s (Figure 1(c)). However, no change in pial collateral vessel size was noted. The lack of enlargement of these vessels could be due to the short duration of LIFU treatment and indicates neuroprotection is likely linked to the vasodilation of smaller caliber capillaries rather than growth of the pial collateral arterioles.

Histological findings confirm microvascular changes in the penumbra of transcranial LIFU-treated mice compared to mock treatment. Although not evaluated in the current study, previous work has highlighted the importance of blood-brain barrier (BBB) permeability in tissue damage following stroke [23]. Our findings suggest that the use of transcranial LIFU at a fundamental frequency of 1.1 MHz and a peak negative pressure of 2.0 MPa for 30 minutes after ischemic stroke at defined pulsing parameters can mediate tissue protection compared to previous studies. Indeed, issues have been raised regarding the use of low-frequency ultrasound causing increased blood-brain barrier disruptions and intracerebral hemorrhage—particularly in the presence of recombinant tissue plasminogen activator—which was corroborated in rat experiments using 20 kHz ultrasound [24]. We also observed cortical hemorrhagic lesions that appeared like traumatic cerebral contusions on gross morphology and increased mortality when increased ultrasonic pressures were applied to subjects using the same FUS system and pulse schema, but at a peak negative pressure of approximately 3.5 MPa.

Our findings demonstrate the critical need for extensive preclinical evaluation of new devices that can deliver optimal ranges of transcranial LIFU as a therapeutic strategy for ischemic stroke. In this study, we show that our 1.1 MHz system was capable of delivering transcranial LIFU in order to safely induce significant neuroprotection when applied for 30 minutes after the onset of ischemic stroke. Further studies are needed to test the therapeutic window, for example, application up to 6-8 hrs postonset of stroke, using large animal models to adjust for skull thickness, as well as uncovering the cellular and molecular mechanisms underlying neural tissue protection that may include microvascular remodeling. Additional modes of action may include modulation of BBB, edema, vasospasms, tissue permeability, interstitial flow, and innate immune regulation [22, 25–27]. Overall, this work demonstrates the neuroprotective properties of LIFU in a murine model of stroke and provides insight into its possible role as a novel neural therapeutic for the treatment of cerebral ischemia.

## 4. Methods

### 4.1. Animals

All rodents were bred and housed in an AAALAC accredited, virus/antigen-free facility with a 12 hrs light-dark cycle; food and water were provided *ad libitum*. Male CD1 mice ages 8-12 weeks were purchased from Charles River, Durham, NC. All mice were given defined codes prior to pMCAO surgery to enable double-blinded experimentation. A total of fourteen mice were used for the initial safety testing, and 23 mice were used for the final study.

### 4.2. Surgical Procedures

The murine model of pMCAO was performed as previously described. Briefly, 8-12-week old male mice were given Buprenorphine-SR (0.15 mg/kg, ZooPharm, Laramie, WY); then, anesthesia was induced using 2% isoflurane-30% oxygen. During the procedure, mice were maintained at 37±0.5°C and monitored for changes in respiratory rate. The skull was thinned to expose and cauterize the main and two distal branches of the left middle cerebral artery (MCA). Sham mice received identical procedures and skull thinning, without ligation using cauterization. Transcranial FUS was performed immediately following pMCAO procedures for 30 minutes as detailed below. For mock LIFU-treated animals, the mice were placed under the transducer for 30 minutes, but the ultrasound system was not turned on. During the procedure and recovery, mice were monitored for signs of distress and hemorrhage. Mice were euthanized by vessel painting at 24 hours post-pMCAO.

### 4.3. Focused Ultrasound System and Treatment Parameters

The transcranial focused ultrasound (FUS) system used in this study is depicted in Figure 1. Two connected function generators were controlled via a current-generation data acquisition driver (NI USB-6501, National Instruments Corp., Austin, Texas, USA) interfacing with a custom MATLAB (The MathWorks, Natick, MA, USA) script using the Data Acquisition Toolbox (MATLAB Data Acquisition Toolbox, The MathWorks, Inc., Natick, MA, USA). MATLAB was used to trigger the first function generator by sending a current signal to the generator at the interstimulus interval (ISI=3 s) for 600 stimulations, resulting in a total sonication time of 30 minutes. The first function generator (SDG5082, Siglent Technologies, Solon, OH, USA) generated a square wave used to trigger the second function generator (SDG1025, Siglent Technologies, Solon, OH, USA) and controlled the pulse repetition frequency (PRF=1 kHz), the duty cycle per pulse (DC=50%), and the sonication duration (SD=300 ms). The second function generator was used to control the ultrasound fundamental frequency (FF=1.1 MHz), the tone-burst duration (TBD=0.5 ms), and the peak negative pressure (*p*−). All pulsing parameters were chosen after a thorough review of published studies investigating LIFU for neuromodulation and neuroprotection ([17, 28]; L. [29, 30]). The pulsed signal from the second function generator was amplified by a linear power amplifier (2100 L, Electronics and Innovation, LTD, Rochester, NY, USA) and transmitted to a 1.1 MHz single element focused ultrasound transducer with estimated focal dimensions of 10.21 mm in length and 1.37 mm in diameter (H-101, Sonic Concepts, Inc., Bothell, WA, USA) through its corresponding matching network (Sonic Concepts, Inc., Bothell, WA, USA). The output from both function generators was verified and monitored in real time during treatment using a 2-channel oscilloscope (Handyscope HS5, TiePie engineering, Sneek, Friesland, The Netherlands) and Multi-Channel Software (TiePie Multi Channel Version 1.42.3.1000/0.9.7.3, TiePie engineering, Sneek, Friesland, The Netherlands). The FUS ultrasound transducer was coupled to the mouse skull using a conical coupling device (C-101, Sonic Concepts, Inc., Bothell, WA, USA) filled with degassed water. Ultrasound coupling gel was placed between the membrane of the coupling cone and the animal’s skull to further reduce signal attenuation. The components of the FUS system are summarized in Figure 1(a), and a picture of this setup is shown in Figure 1(b). The pulsing regime employed in these studies is pictured in Figure 1(c).

Transcranial FUS was applied to each subject immediately following the pMCAO cauterization procedure (i.e., stroke) for 30 minutes using the pulsing parameters listed above. Focal pressures were measured prior to experiments using a high-sensitivity calibrated rod hydrophone (HNR-0500, Onda Corp., Sunnyvale, CA, USA). Before treatment, the FUS transducer was positioned over on the lest parietal bone 4 mm rostral of bregma and 2 mm lateral of midline (sagittal suture) on all treated mice and two studies were completed. First, a safety study was conducted on fourteen adult, male CD1 mice with experimental groups as follows. Three sham mice (i.e., mice with craniotomy but without MCA ligation) were treated with 30 minutes of LIFU therapy at *p*− -1.8 MPa, three sham mice were treated with LIFU at *p*− -3.5 MPa, and two sham mice were left untreated. Four pMCAO (i.e., stroke) mice were also treated with LIFU at *p*− 3.5 MPa, and two pMCAO mice were left untreated. Mice included in the pilot study were monitored for the appearance of gross hemorrhage in the treatment area immediately after treatment and for 24 hours posttreatment. Mortality of each group was also recorded.

Based on the results of the pilot study, eleven adult, male CD1 mice were subjected to the LIFU treatment at a peak negative pressure of -2 MPa. Twelve additional mice were used as an experimental control by applying mock ultrasound where the FUS transducer was positioned over the same region as the treated mice, and anesthesia was maintained for 30 minutes without turning on the ultrasonic device.

### 4.4. Vessel Painting and Pial Collateral Quantification

The vessel painting technique was performed as previously described [15, 31]. Briefly, male mice were injected with heparin (2,000 units/kg) and sodium nitroprusside (SNP, 0.75 mg/kg) five minutes prior to euthanization, using an overdose of isoflurane. Mice were then perfused with 10 ml of 1X phosphate buffered saline (1X PBS) containing 20 units/ml heparin and then 10 ml DiI (0.01 mg/ml, Invitrogen) -4% sucrose-PBS-heparin mixture at a flow rate of 2 ml/min followed by 50 ml of cold 4% paraformaldehyde (PFA). Fixed brains were imaged and tiled at 4x magnification on an inverted Nikon C2 confocal microscope. Images were imported into ImageJ (NIH) for quantification of the number and diameter of intercollaterals, as described [13, 16].

### 4.5. Infarct Volume, IgG Deposition Volume, and CD31 Immunostaining

Perfused fixed brains were cryopreserved in 25% sucrose and then embedded in OCT and serial cryosectioned. Infarct volume (mm^3^) was assessed by the Cavalieri Estimator probe using the StereoInvestigator software (MicroBrightField, Williston, VT, USA), as previously described. Briefly, six serial coronal sections, cut at 30 *μ*m, were stained using a 0.2% Cresyl violet solution (Electron Microscopy Science, Hatfield, PA) or anti-mouse IgG-488 (Invitrogen, Waltham, MA). The total volume of infarct was quantified by estimating the area of tissue loss in the ipsilateral cortical hemisphere using six, serial coronal sections. A 100 *μ*m spaced grid was placed over the ipsilateral hemisphere in the Cavalieri probe and infarcted area scored. The coronal sections were used for immunohistochemical (IHC) staining of CD31. Sections were blocked with 2% cold water fish skin gelatin (Sigma, Inc., St. Louis, MO) in 0.2% Triton-X100, incubated with goat anti-CD31 (1 : 100, R&D systems, Minneapolis, MN, USA) overnight at room temperature (RT), washed with 1X PBS, and then incubated with donkey anti-goat 488 (1 : 250, Thermofisher, USA) for 1 hour at RT. Slides were then mounted with DAPI counterstain (SouthernBiotech, Birmingham, AL), and images were acquired using Olympus fluorescence microscope. Microvessel diameters were measured using ImageJ.

### 4.6. Statistical Analysis

Data was graphed using GraphPad Prism, version 9 (GraphPad Software, Inc., San Diego, CA). Where appropriate, Student’s two-tailed t-test was used for comparison of two experimental groups and multiple comparisons by one-way or two-way ANOVA followed by *post hoc* Bonferroni test. Changes were identified as significant at ${}^{*}P<0.05$, ${}^{**}P<0.01$, and ${}^{***}P<0.001$. Each mean value was reported together with the standard error of mean (SEM). An experimenter blinded to the conditions performed all lesion volume and IgG deposition quantifications.

### 4.7. Study Approval

All procedures were conducted in accordance with the NIH Guide for the Care and Use of Laboratory Animals, the Virginia Tech Institutional Animal Care and Use Committee (IACUC; #18-088).

## Data Availability

The datasets generated during and/or analyzed during the current study are available from the corresponding author on reasonable request.

## Abbreviations

VP: Vessel painting
pMCAO: Permanent middle cerebral artery occlusion
LIFU: Low-intensity focused ultrasound.
